# 3D Architectured Graphene/Metal Oxide Hybrids for Gas Sensors: A Review

**DOI:** 10.3390/s18051456

**Published:** 2018-05-07

**Authors:** Yi Xia, Ran Li, Ruosong Chen, Jing Wang, Lan Xiang

**Affiliations:** 1 Research Center for Analysis and Measurement, Kunming University of Science and Technology, Kunming 650093, China; 2 The Key Laboratory of Unconventional Metallurgy, Ministry of Education, Kunming 650093, China; 3 Department of Chemical Engineering, Tsinghua University, Beijing 100084, China; crs15@mails.tsinghua.edu.cn; 4 Faculty of Environmental Science and Engineering, Kunming University of Science and Technology, Kunming 650093, China; ranli0904@163.com; 5 The Key Laboratory of Food Colloids and Biotechnology, Ministry of Education, School of Chemical and Material Engineering, Jiangnan University, Wuxi 214122, China

**Keywords:** 3D architectured hybrids, graphene, metal oxide, gas sensor

## Abstract

Graphene/metal oxide-based materials have been demonstrated as promising candidates for gas sensing applications due to the enhanced sensing performance and synergetic effects of the two components. Plenty of metal oxides such as SnO_2_, ZnO, WO_3_, etc. have been hybridized with graphene to improve the gas sensing properties. However, graphene/metal oxide nanohybrid- based gas sensors still have several limitations in practical application such as the insufficient sensitivity and response rate, and long recovery time in some cases. To achieve higher sensing performances of graphene/metal oxides nanocomposites, many recent efforts have been devoted to the controllable synthesis of 3D graphene/metal oxides architectures owing to their large surface area and well-organized structure for the enhanced gas adsorption/diffusion on sensing films. This review summarizes recent advances in the synthesis, assembly, and applications of 3D architectured graphene/metal oxide hybrids for gas sensing.

## 1. Introduction

With the rapid development of modern industry, the detection of hazardous gases has become an important issue for human health and environmental protection. A variety of materials such as carbon-based materials, noble metals, metal oxides or sulfides and organic semiconductors have been explored to fabricate gas sensors [1,2,3,4,5,6,7,8,9,10,11,12,13,14,15]. Among them, graphene, including reduced graphene oxide (rGO)/metal oxide-based hybrid-structures, has been proved as a potential sensing material for gas sensors due to its low temperature sensitivity and fast carrier transportation properties [16,17,18,19,20,21,22,23,24,25]. 

In the past decades, graphene has received more and more attention for application in gas sensors because of its high electrical conductivity, surface area (2630 m^2^/g), and charge carrier mobility (15,000 cm^2^·V^−1^·s^−1^) at room temperature [16,19,26,27,28]. The superiority of graphene for gas sensing relies on two basic factors associated with its 2D dimensions, i.e., the ultrahigh surface area per atom and high electron transport along the graphene base-plane. However, further efforts are required to solve some issues, e.g., the insufficient sensitivities, long dynamic responses, poor repeatability and selectivity of rGO-based gas sensors [10,25,27,28,29]. On the other hand, graphene exhibits excellent anchoring ability as a substrate for chemical functionalities or nanomaterials and, thus, fabrication of novel graphene-based nanohybrids has been an effective approach for improving the gas sensing properties [30]. Metal oxide (MOx) semiconductors represent promising materials for gas sensing because of their high sensitivity, selectivity to gas molecules, good stability, low cost and various controllable nanostructures [31,32,33,34,35,36,37,38,39,40]. Hence, graphene/MOx-based nanohybrids have been identified as promising candidates for gas sensing application due to their enhanced sensing performance and synergetic effects between the two components. Numerous MOx such as SnO_2_, ZnO, WO_3_, etc. were utilized to form hybrids with graphene for improving the gas sensing properties [41,42,43,44]. Nevertheless, as sensing materials graphene/metal oxide nanocomposites still have several limitations. For example, the sensitivity and response rates are still insufficient; illumination or thermal treatment is required for recovery in some sensors [41,42,43,44,45,46] so the construction of highly sensitive and rapid-response graphene/MOx nanohybrid-based gas sensors still remains a challenge.

3D hierarchical structures have recently attracted much attention for the synthesis of gas sensors owing to their large surface area and well-organized porous structure which improves the gas adsorption/diffusion on sensing films [47,48,49,50]. Thus, researchers have been shifting their interest to the construction of well-formed 3D graphene or reduced graphene oxide (rGO)/MOx hybrids for highly sensitive, selective and cost-effective gas sensors. Up to now, several reviews have been published on the design of rGO/MOx-based nanostructures for gas sensors with various morphologies [25,51,52,53,54,55]. However, only a small part of these studies were aimed at the construction of 3D graphene/MOx nanohybrids for gas sensing applications. Herein, we present a review on 3D architectured graphene/metal oxide hybrids for gas sensors. First, various combination strategies for preparing different 3D rGO/MOx nanostructures are reviewed, including composites combining 2D or 3D graphene with dimensionally different metal oxides such as nanorods, nanosheets, and hierarchical structures, etc. Then, gas sensing applications of 3D architectured graphene/MOx hybrids, especially the development of gas detection at room temperature, are discussed.

## 2. Construction of 3D Graphene/MOx Nanostructures

### 2.1. 2D Graphene/1D MOx Based Architectures

One dimensional metal oxides show many advantages in gas sensing applications because of their high surface-to-volume ratio, abundant surface states and potential to assemble hierarchical structures [9]. Nanostructures such as nanotubes, nanowires, nanorods, and nanofibers have been widely utilized for the construction of 3D hybrid sensing materials with graphene under template-assisted or multistep sequential growth synthesis conditions.

#### 2.1.1. Template-Assisted Synthesis

**Figure 1:**
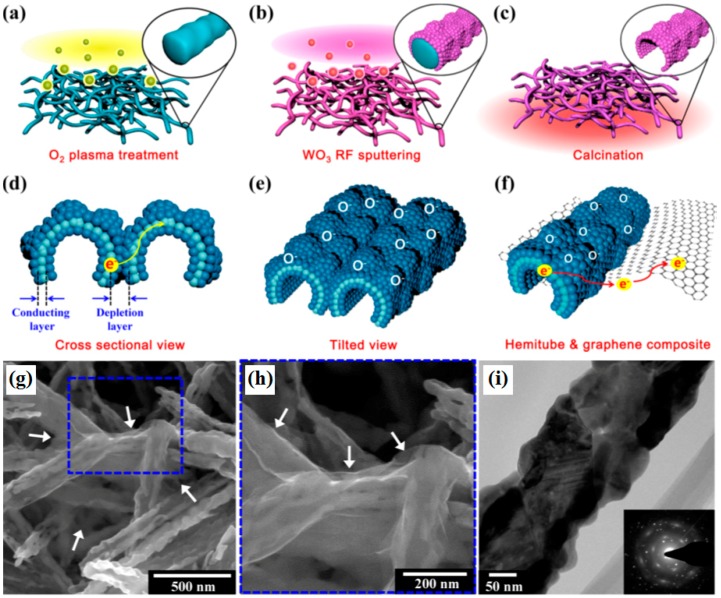
(**a**–**e**) Schematic illustration of formation of 3D graphene-WO_3_ hemitube architectures; (**g**–**i**) SEM and TEM image with SAED pattern in the inset of graphene-WO_3_ hemitube composite. Reproduced with permission from [57], © 2012 American Chemical Society

Template-assisted methods have been widely used in fabricating 3D architectures, due to the advantages of the diverse morphology of the available templates and large-scale synthesis [56]. Choi et al. reported 3D WO_3_ hemitubes functionalized by graphene with high surface area made using a nonwoven polymeric fiber composed of polyvinylpyrollidone (PVP)/poly(methyl methacrylate) (PMMA) composite as template under O_2_ plasma treatment conditions [57]. A schematic illustration of the graphene/WO_3_ 3D structure formation process is shown in Figure 1a–f. Firstly, WO_3_ hemitube structures with wrinkled, bumpy surface topology were achieved by RF-sputtering WO_3_ films onto the O_2_ treated PVP/PMMA composite nanofiber templates, followed by high temperature calcination to remove the polymeric template; Finally, graphene was homogenously mixed with WO_3_ hemitubes to form 3D nanocomposites owing to the heterojunction between WO_3_ hemitubes and graphene induced by the charge transportation [57]. The morphology of 3D graphene/WO_3_ hemitubes is showed in Figure 1g–i.

#### 2.1.2. Multistep Sequential Growth 

Although template-based methods have been one of the most promising routes to fabricate 3D hybrids, some problems such as tedious experimental procedures, the high cost of templates, and residual impurities have limited its development in application [58]. Therefore, convenient and efficient multistep approaches have been developed to produce many desired 3D hybrid structures [59].

Deng et al. developed 3D rGO-conjugated Cu_2_O-nanowire mesoporous hybrids in the presence of graphene oxide (GO) and *o*-anisidine in a one-pot hydrothermal treatment [60]. The mesocrystals consisted of highly anisotropic nanowires as building blocks and possessed a distinct octahedral morphology with eight {111} equivalent crystal faces [60].

**Figure 2:**
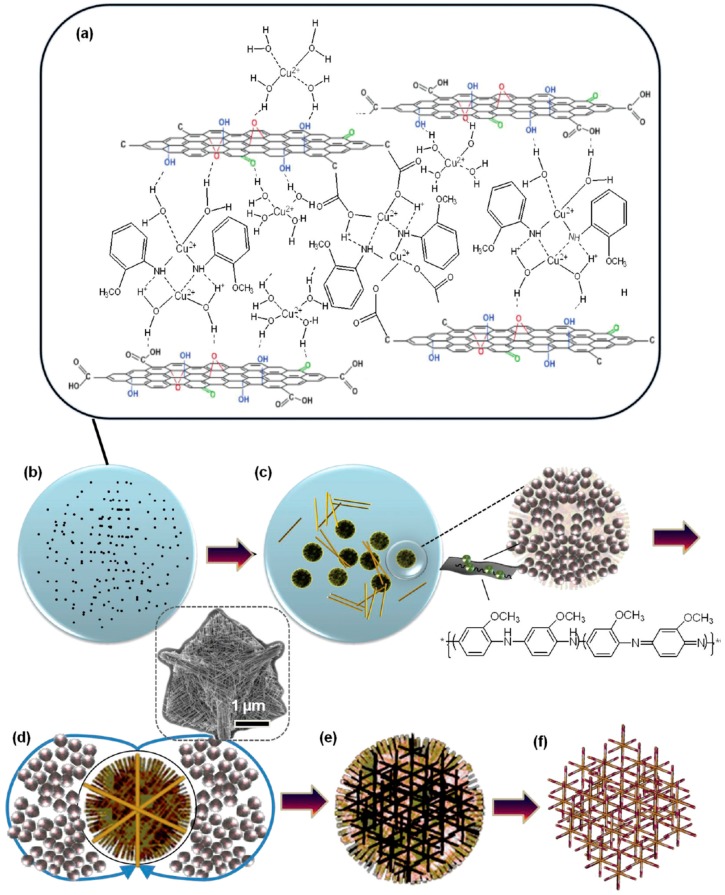
(**a–f**) Schematic illustration of the Cu_2_O crystallization process assisted by o-anisidine and GO. Reproduced with permission from [60], © 2012 American Chemical Society.

The multistep sequential growth mechanism of the mesoporous hybrids is as follows (Figure 2a–f): Firstly, GO-induced agglomeration of amorphous spherical Cu_2_O nano particles at the primary stage resulting in the transition of a growth mechanism from conventional ion-by-ion growth to particle mediated crystallization; then, the formed amorphous microspheres developed into hierarchical mesoporous nanowire assemblies through mesoscale transformation by Ostwald ripening; finally, the porous 3D framework structures interspersed among 2D rGO sheets leading to the self-organization of large-scale 3D mesoporous hybrid architecture where the GO was reduced simultaneously [60]. 

**Figure 3:**
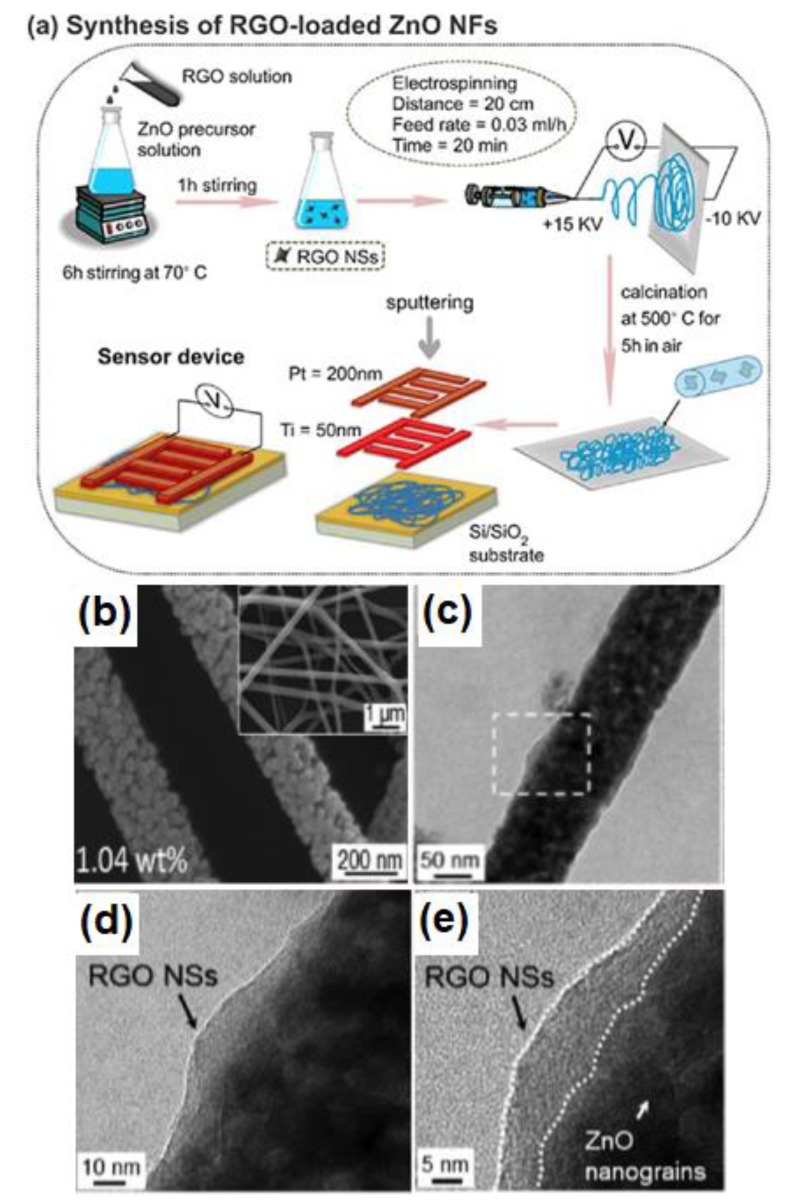
(**a**) Schematic illustration of formation of rGO nanosheet-loaded ZnO core-shell nanofibers; (**b**–**e**) SEM and TEM pictures of samples. Reproduced with permission from [61], © 2015 Elsevier.

3D core-shell rGO/MOx structures can also be achieved through a multistep strategy. For example, Abideen et al. fabricated rGO nanosheet-loaded ZnO core-shell nanofibers using a simple electrospinning method [61]. The two-step formation process is shown in Figure 3a. Firstly, the precursor solution was obtained by mixing a Zn^2+^-containing solution with rGO for a certain time, leading to the formation of the ZnO/rGO precursor. Then, the 3D rGO/ZnO core-shell nanofibers were formed under electrospinning treatment. The morphology of as-prepared samples is shown in Figure 3b–d. A similar strategy has also been used to conjugate rGO with SnO_2_, In_2_O_3_, Fe_2_O_3_ and so on [62,63,64]. 

**Figure 4:**
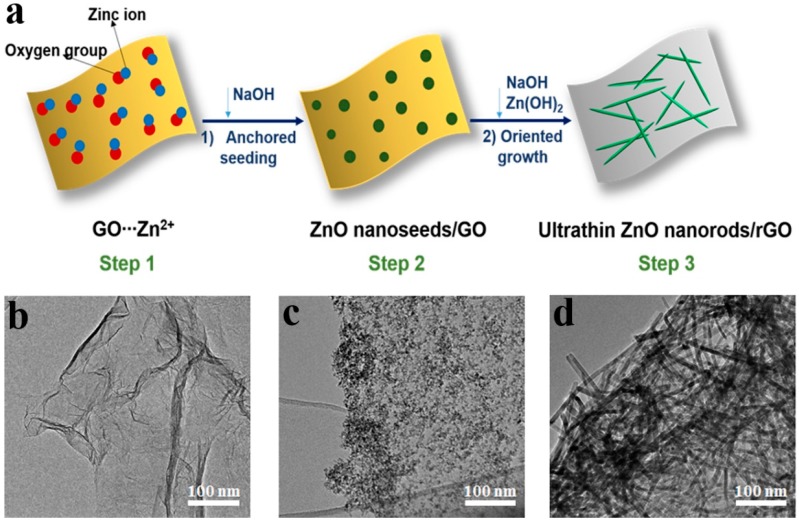
(**a**) Schematic illustration of fabrication of rGO/UT-ZNR; (**b–d**) TEM images of GO, GO/ZnO nanoseeds and rGO/UT-ZNR. Reproduced with permission from [65], © 2016 American Chemical Society.

We recently successfully developed a facile and efficient two-step solution method to synthesize 3D mesoporous rGO/ultrathin ZnO nanorods nanocomposites (rGO/UT-ZNR) in 10 min at 80 °C [65]. rGO/UT-ZNR were obtained via the in-situ growth of ZnO nanoseeds on GO nanosheets followed by oriented growth of the nanoseeds into ZnO nanorods in a Zn(OH)_2_/NaOH mixed suspension (Figure 4a). We firstly prepared the Zn(Ac)_2_ methanol solution added with GO under continuous stirring to achieve adsorption equilibrium. Zinc ions were firmly absorbed on the surface of GO nanosheets owing to the strong metal ion anchoring ability of the functional groups from GO (step 1). After that, ZnO nanoseeds layer was formed on the GO nanosheets in NaOH methanol solution (step 2). Then, the as-prepared GO/ZnO nanoseeds mixture was added to an aqueous suspension containing NaOH/Zn(OH)_2_ precursor for the further evolution of the ZnO nanoparticles into ultrathin ZnO nanorods, while the in-situ reduction of GO to rGO occurred simultaneously (step 3) [65].

In our case, GO nanosheets not only evolved into rGO in the nanohybrids, but also provided a confined space for ion adsorption, anchored nucleation and subsequent growth of ZnO nanorods, resulting in the formation of 3D rGO/UT-ZNR mesoporous nanohybrids [65]. The morphology of rGO/UT-ZNR is shown in Figure 4d. Furthermore, gram-scale (ca. 1.2 g) 3D rGO/UT-ZNR nanohybrids can be successfully produced after only 10 min of reaction. Such efficient large-scale production of 3D rGO/MOx architectures may allow the opportunity for commercial application as sensing materials.

### 2.2. 2D Graphene/2D MOx Based Architectures

MOx nanosheets are other potential units for the formation of 3D rGO/ZnO hybrids for gas sensors owing to their large surface area [66,67,68,69,70]. However, 2D MOx nanostructures are difficult to hybridize with graphene due to the weaker affinity between them [53,54]. Up to now, just a few studies were reported in this area [71,72,73]. 

**Figure 5:**
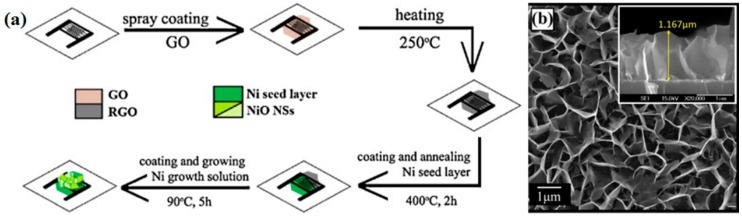
(**a**) Schematic illustration of formation of 3D porous rGO/NiO nanosheets hybrids; (**b**) SEM pictures of samples. Reproduced with permission from [71], © 2013 Elsevier.

For instance, Hoa et al. developed novel 3D porous composites consisting of 2D graphene and 2D NiO nanosheets (NSs) using a low-cost and large area scalable solution-based process at low temperature [71]. The 3D hybrid architectures were obtained in two steps. First, GO films were spray coated on the electrodes and reduced to rGO via a heating treatment. Then Ni seeds were coated and annealed on the rGO films, followed by the formation of NiO nanosheets after the reaction in precursor solution (Figure 5a). The morphology of 3D porous rGO/NiO hybrids is shown in Figure 5b.

### 2.3. 2D Graphene/3D MOx Architectured Hybrids

3D hierarchical structures have been recently attracted much attention for the fabrication of gas sensors because of their larger surface area and well-constructed 3D structures that improve gas adsorption/diffusion on sensing films [47,48,49,50]. Various 3D MOx nanostructures have been successfully used to combine with rGO nanosheets due to the advantage of the easily-controlled morphology of MOx. 

#### 2.3.1. Graphene/Regular 3D MOx Nanostructures

**Figure 6:**
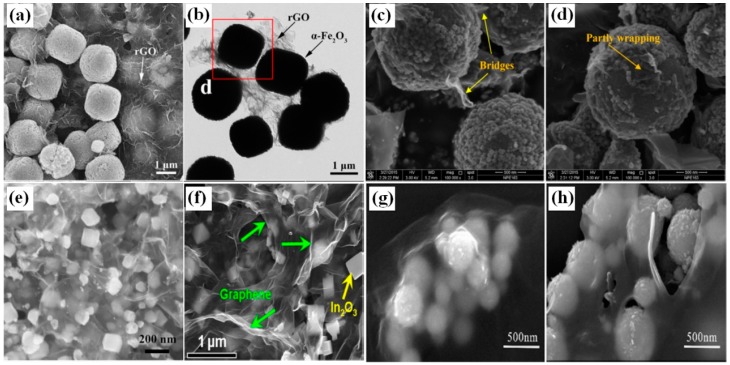
Morphology of (**a–b**) rGO/α-Fe_2_O_3_ cubic nanostructure [74]; (**c,d**) rGO decorated TiO_2_ microspheres [78]; (**e**) N-rGO/NiO cube [75]; (**f**) rGO/In_2_O_3_ cube nanocomposites [76]; (**g,h**) graphene-wrapped WO_3_ nanospheres [79]. (**a–d**) and (**g,h**) were reproduced with permission from [74,78,79], © Elsevier. (**e,f**) were reproduced with permission from [75,76] © American Chemical Society.

Many studies have been focused on the combination of 3D nanostructures such as spheres, cubes and rGO nanosheets because nanostructures with regular shape are more easily covered and connected with the easily crinkled and folded rGO nanosheets [74,75,76,77,78,79,80,81,82]. For example, Zhang et al. demonstrated rGO/α-Fe_2_O_3_ composites with 3D nanostructures using a low-cost and environmentally friendly hydrothermal method [74]. Uniform α-Fe_2_O_3_ cubes adhered uniformly on both sides of the crumpled and rippled rGO sheets (Figure 6a,b) [74]. A novel 3D nitrogen-doped reduced template and surfactant free graphene oxide (N-rGO)/NiO cube (hc-NiO) composite was obtained through a facile hydrothermal method and a post-calcination treatment [75]. The in-situ growth of NiO cubes organized by many nanoparticles on the surface of N-rGO layers can be observed in Figure 6e [75]. rGO/In_2_O_3_ cube nanocomposites were also successfully synthesized by a facile one-step microwave-assisted hydrothermal method (Figure 6f) [76]. 

MOx with spherical structures can be also introduced to form 3D hybrids with rGO nanosheets. For example, rGO decorated TiO_2_ microspheres are obtained under a hydrothermal method [78]. rGO nanosheets play a dual role, in which they not only cover some TiO_2_ balls to form a partially “wrapping” microstructure (Figure 6d), but also act as a “bridge” between two neighboring oxide particles (Figure 6c), leading to the formation of novel 3D rGO-MOx nanostructures. In the other hand, a facile sol-gel method was employed to synthesize 3D graphene-wrapped WO_3_ nanospheres composite [79]. Different from the partially wrapped TiO_2_ spheres, the WO_3_ nanospheres were distinctly enwrapped with gauze-like graphene nanosheets (Figure 6g,h). Besides, Fe_2_O_3_ nanospheres, SnO_2_ hollow particles and SnO_2_ discoid crystal can also be modified by rGO for novel 3D nano-hybrids [80,81,82].

#### 2.3.2. Graphene/3D MOx Hierarchical Assemblies 

To improve the gas adsorption/desorption, 3D MOx hierarchical assemblies organized via building blocks such as nanoparticles, nanorods, and nanosheets have been widely developed by combining rGO owing to their high surface area and porous structures [83,84,85,86,87,88,89,90,91,92]. 

**Figure 7:**
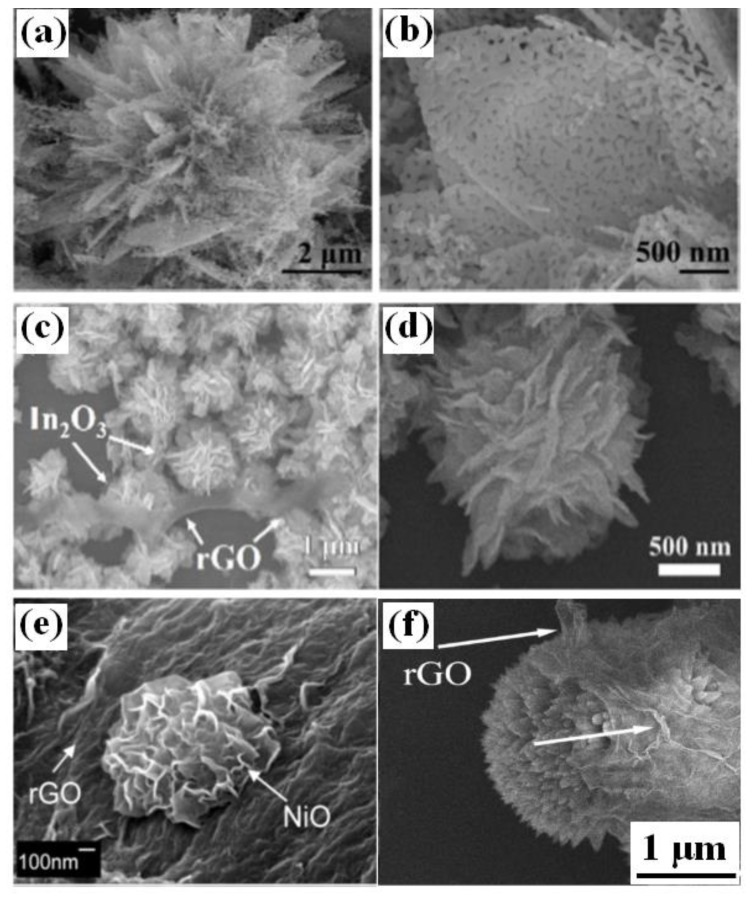
Morphology of (**a,b**) 3D hierarchical porous ZnO nanoflowers modified with rGO [83]; (**c,d**) 3D rGO-In_2_O_3_ composite [84]; (**e**) rGO/NiO nanoflowers [85]; (**f**) urchinlike CuO 3D structures modified by rGO [86]. (**a–e**) were reproduced with permission from [83,84,85], © Elsevier. (**f**) were reproduced with permission from [86] © 2014 American Chemical Society.

Li et al. reported the preparation of novel 3D hierarchical porous ZnO nanoflowers modified with rGO under hydrothermal reaction conditions [83]. The uniform 3D flower-like structures are assembled by nanosheets with porous structures (Figure 7a, b). By utilizing a facile one-step hydrothermal method, Liu et al. fabricated 3D sensing materials composed of hierarchical flower-like In_2_O_3_ and rGO [84]. In the 3D rGO-In_2_O_3_ composite, as shown in Figure 7c, d, flexible and transparent rGO sheets were placed among flower-like hierarchical In_2_O_3_ organized by nanosheets [84]. Ngo et al. also developed 3D hybrids in which NiO nanoflowers were uniformly grown on the surface of rGO by a facile hydrothermal method followed by annealing under flowing nitrogen (Figure 7e) [85]. 

Besides these nanosheet-based 3D nanohybrids, rGO/nanorod-assemblybased 3D nanostructures have been also developed [86,87,88]. For example, urchin-like CuO 3D structures modified by rGO were fabricated by a one-pot microwave-assisted hydrothermal method [86]. The connection between CuO and rGO can be observed and rGO shows a crumpled layered structure distributed randomly in the composites with some stacking layers (Figure 7f) [86]. 

On the other hand, template-induced porous MOx structures can also be coupled with rGO to form 3D composites [89,90]. Xue successfully produced 3D ordered mesoporous In_2_O_3_-rGO nanocomposites using mesoporous silica as a hard template through ultrasonic mixing [90]. Zhu et al. employed a facile method to obtain rGO/SnO_2_ 3D microporous nanocomposites by a simple blending and deposited onto different microporous substrates [91]. 

#### 2.3.3. Graphene-MOx Based Ternary 3D Hybrids 

**Figure 8:**
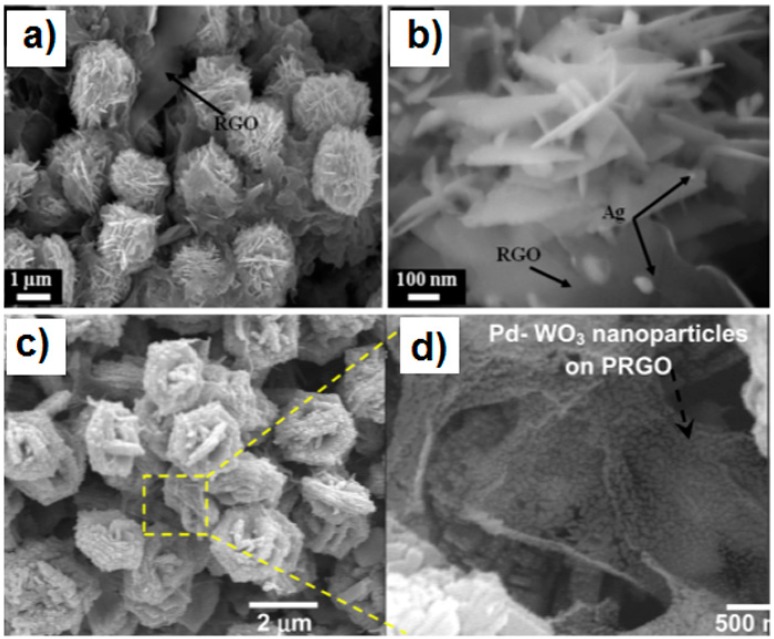
Morphology of (**a,b**) Ag-loaded 3D ZnO nanostructure-rGO [93]; (**c,d**) 3D rGO-In_2_O_3_ composite [94]. Reproduced with permission from [93,94], © 2015 and 2014 Elsevier.

Single elements such as noble metals can be used to decorate the as-prepared rGO-MOx 3D architectures to form a ternary composite. Uddin et al. synthesized an Ag-loaded 3D ZnO nanostructure-rGO (Ag/ZnO Hrc-RGO) hybrid using a facile hydrothermal method followed by an efficient photochemical route for the Ag deposition [93]. The pH level adjusted by the capping agent molecules (NH_4_OH) and the anisotropic growth of ZnO play very important roles in the formation of hierarchical ZnO microsphere-like nanosheet assemblies. Figure 8a, b show that small-sized Ag nanoparticles with an average particle size of 40 nm are attached onto the ZnO nanosheets, closely affixed onto the 3 to 5-layer thick RGO sheets [93]. Using a controlled hydrothermal process, Esfandiar et al. prepared 3D rGO-WO_3_-Pd ternary composites in which Pd/WO_3_ nanostructures were incorporated on partially reduced graphene oxide (PRGO) sheets [94]. The nanostructure growth of WO_3_ on the graphene sheet could be improved by the addition of the PRGO during the hydrothermal process and the final ternary hybrids exhibited a hierarchical nanostructure with a high surface area (Figure 8c, d) [94].

### 2.4. Graphene-MOx Hybrids Assembled within 3D Graphene-Multilayer Network

**Figure 9:**
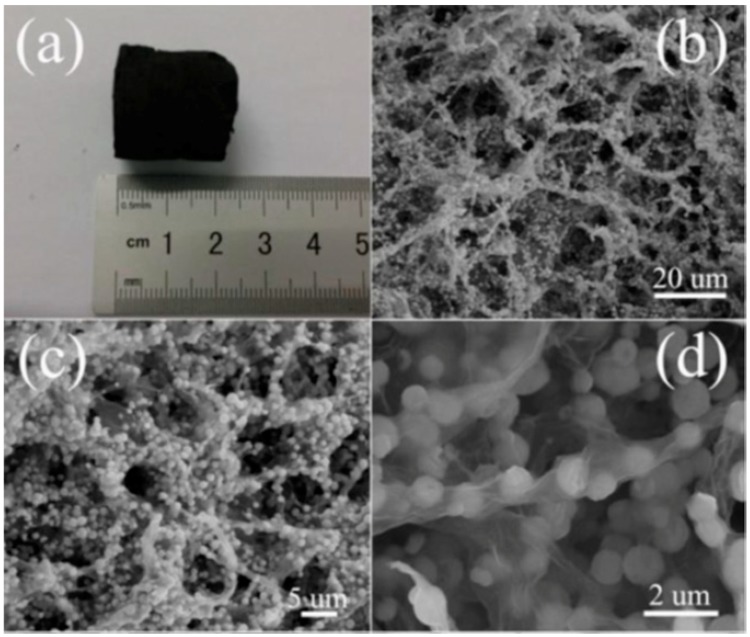
Morphology of 3D rGO aerogel/ZnO spheres composite. (**a**) A photograph of composite. (**b–d**) SEM images with different magnifications of rGO aerogel/ZnO spheres composite. Reproduced with permission from [43], © 2015 Elsevier.

Construction of multilayer networks for GO can serve as a hierarchical space for the growth of MOx to form 3D architectured rGO/MOx hybrids [43,44,95,96,97,98,99,100]. Typically, a 3D rGO aerogel/ZnO spheres composite is produced via a facile solvothermal method [43]. The formation process is described as follows: firstly, ZnCl_2_ was dispersed into multilayer GO solution for ion anchoring. Second, NaNO_3_ and NaAc were added to the mixed solution resulting in a precursor solution. Then, the obtained mixture was transferred to a Teflon-lined stainless-steel autoclave for the in-situ growth of the ZnO spheres under solvothermal treatment conditions, while the *in-situ* reduction of GO to rGO occurred simultaneously. Finally, a black integrated 3D graphene aerogel–ZnO was obtained via a freeze-drying process to maintain the 3D monolithic architecture [43]. The 3D rGO exhibits interconnected macroporous structures and the ZnO spheres featured a size of 0.5–1 μm are anchored homogeneously on the surface of rGO layers (Figure 9a–d).

## 3. Applications of 3D Graphene/MOx Hybrids for Room Temperature Gas Sensing

3D architectured graphene/MOx nanocomposites have been widely used for the fabrication of gas sensors to detect various hazardous gases such as NO_2_, NH_3_, HCHO, H_2_S and so on [101,102,103,104,105]. However, many 3D graphene/MOx-based gas sensors require high operating temperatures (typically >100 °C) to achieve high sensitivity and fast response. The high temperature operation brings issues such as high energy consumption and the risk of gas explosions. These limitations, therefore, have recently motivated the development of high-performance room-temperature gas sensors. NO_2_ seems to be the most investigated room temperature sensing target, not only because its toxicity, but also its electrophilic characteristics that improve the gas chemical adsorption reaction on the surface of sensing materials. Many efforts have been dedicated to control the structures of 3D rGO/MOx sensing materials for enhanced NO_2_ sensing properties at room temperature [65,76,79,80,84,90,92,106,107,108]. 

The synergetic effects between graphene and MOx are the key factors for improving the room temperature NO_2_ sensing performance. In graphene/MOx composites, the MOx nanostructures act as key sensitive materials for the chemical adsorption of NO_2_; on the other hand, the highly conductive graphene can not only ensure the current flow across electrodes for fast response, but also conjugate with MOx to form Schottky-junctions enhancing the electron capture. Thus, many efforts have been dedicated to optimizing the components’ structures in 3D rGO/MOx hybrids to achieve enhanced sensing performance. 

**Figure 10:**
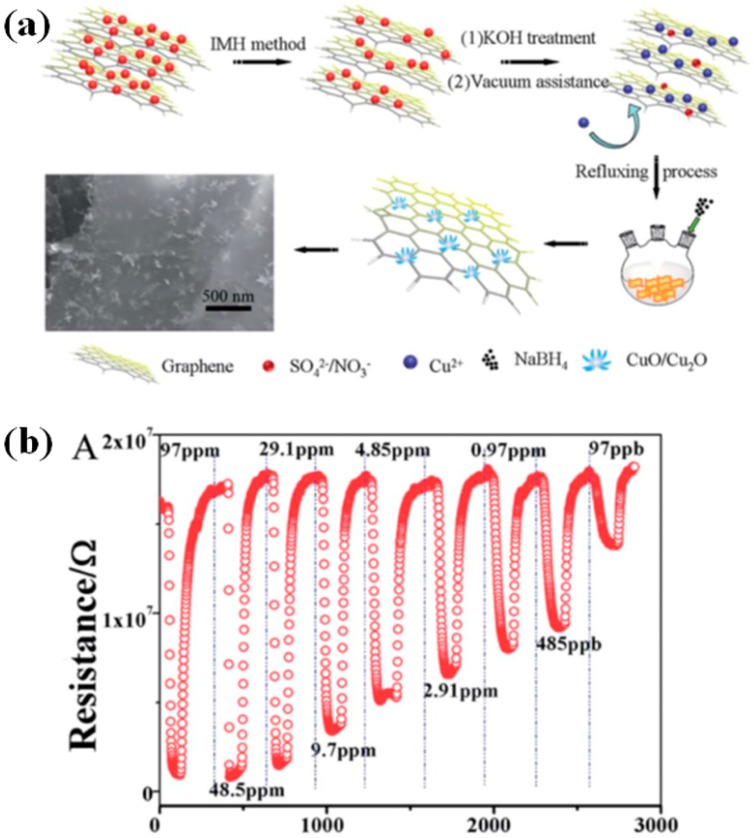
(**a**) Schematic illustration of formation of 3D nanoflower-like CuxO/multilayer graphene; (**b**) Dynamic sensing property of CuMGC at room temperature. Reproduced with permission from [92] © 2014 Royal Society of Chemistry.

Controllable preparation of MOx with confined sizes in the nanohybrids could be one efficient way to contribute to enhancing sensing properties. Yang et al. developed a 3D nanoflower-like CuxO consisting of 5–9 nm ultrafine nanoparticles/multilayer graphene (CuMGC) composites as a room temperature NO_2_ gas sensor [92]. The 3D nanoflower-like CuxO was located in-situ on the multilayer rGO via three steps (Figure 10a). The 3D hybrids showed a high sensitivity (95.1) and fast response time (9.6 s) to 97 ppm of NO_2_ (Figure 10b). The enhanced sensitivity is attributed to the small size 3D CuxO flowers with high surface area which could provide more active sites for the surface adsorption reaction of NO_2_, while the fast response is due to the Schottky contact between rGO and small size CuxO leading to formation of many more donors to capture and migrate electrons from the conduction band. Using a similar route, Mao et al. prepared a 3D rGO-based NO_2_ sensor with enhanced sensitivity (287% to 100 ppm of NO_2_) and selectivity by decoration of ultrafine (3–6 nm) SnO_2_ nanocrystals [108]. 

The design of porous structures was also proved to be feasible route to enhance the performance of 3D rGO/MOx nanohybrids for room temperature NO_2_ sensing. For instance, a gas sensor based on 3D mesoporous rGO aerogels embedded with SnO_2_ or ZnO showed an enhanced response rate (190 s and 200 s to 10 and 100 ppm of NO_2_) [43,44]. However, the low sensitivity (<10%) limited the practical application of those gas sensors. 

**Figure 11:**
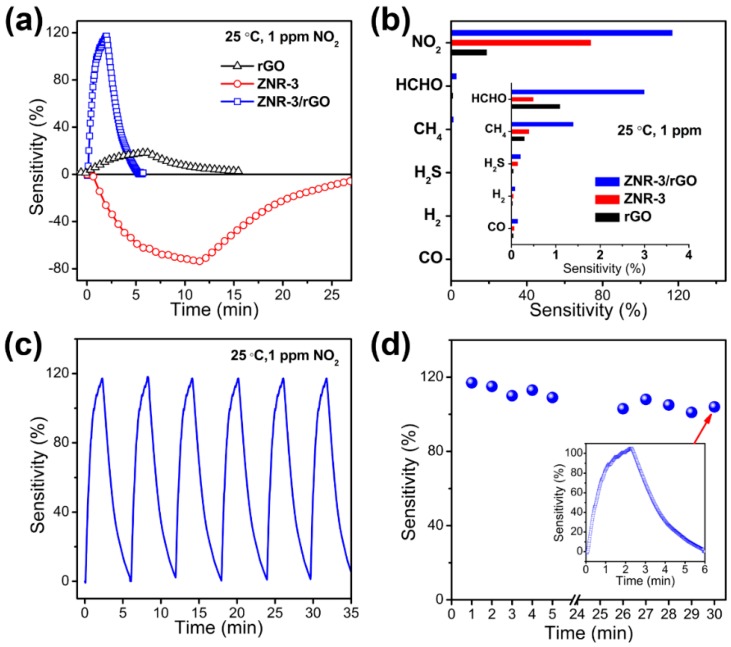
(**a**) dynamic response of rGO, UT-ZNR, and rGO/UT-ZNR; (**b**) sensitivities rGO, UT-ZNR, and rGO/UT-ZNR; (**c**) reproducibility of the rGO/UT-ZNR sensor; (**d**) stability of the rGO/UT-ZNR sensor in air for 30 days. Reproduced with permission from [65] © 2016 American Chemical Society.

We recently developed a novel high-performance room-temperature NO_2_ sensor based on 3D rGO/ultrathin ZnO nanorods (rGO/UT-ZNR) with simultaneous characteristics of size-confined ZnO nanorods (with average diameter of 12 nm) and mesoporous structure [65]. The rGO/UT-ZNR-based NO_2_ sensor exhibited high sensitivity (119% to 1 ppm NO_2_), low LOD of 50 ppb and fast response and recovery (75 and 132 s to 1 ppm NO_2_). Meanwhile, the rGO/UT-ZNR sensor presented a favorable linearity, good selectivity and stability (Figure 11a–d). The 3D rGO/UT-ZNR nanohybrids with high surface area and mesopore structures improved the of NO_2_ diffusion/adsorption within the nanohybrid film, resulting in a higher sensitivity and faster sensing dynamics than rGO- or UT-ZNR- based sensors at room temperature [65]. On the other hand, the selective adsorption and strong adsorption capacity between ZnO and NO_2_ induced the outstanding selectivity of the rGO/UT-ZNR nanohybrids.

**Figure 12:**
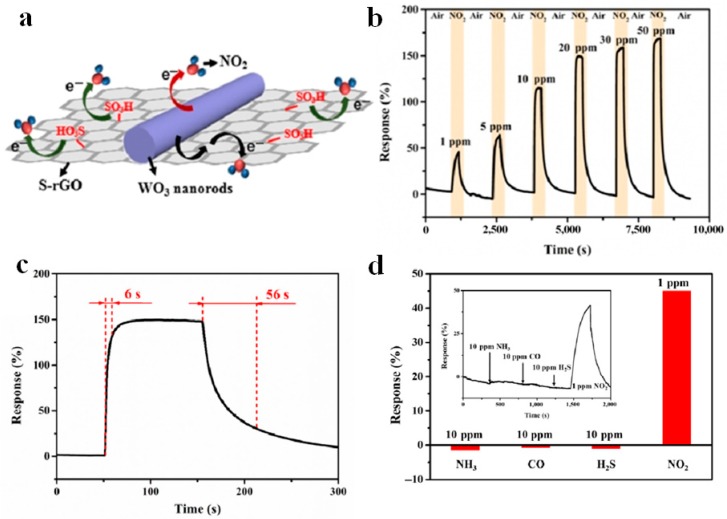
(**a**) Schematic illustration of NO_2_ adsorption on S-rGO/WO_3_ surface; (**b**,**c**) Dynamic response of the 3D S-rGO/WO_3_ hybrids of NO_2_ at room temperature.; (**d**) Selectivity of 3D S-rGO/WO_3_. Reproduced with permission from [106] © 2018 Springer.

Decorating MOx using functionalized rGO has been seen as another effective route to establish 3D rGO/MOx-based gas sensors with high performance [109]. Wang et al reported the synthesis of sulfonated rGO (S-rGO)/WO3 nanorod 3D composites via a simple and cost-effective hydrothermal method (Figure 12a) [106]. 

In this case, sensing performance towards 1–50 ppm has been demonstrated (Figure 12b). The optimized S-rGO/WO_3_-based sensor showed a high and fast (6 s) sensitivity (149%) to 20 ppm NO2 and a short recovery time (56 s) (Figure 12c). Furthermore, enhanced reproducibility, selectivity, and fast recovery kinetics can be also observed (Figure 12d). The enhanced sensing mechanism can be ascribed to the following three factors: firstly, the Schottky-junction formed in S-rGO/WO3 nanocomposites could provide more active sites to capture electrons. Then, the chemical bonding achieved via C–O–W bonds formed between S-rGO and WO3 can be regarded as a charge transport bridge in the gas sensing process, which significantly enhances the charge transfer efficiency, resulting in the high sensitivity and fast response [79,106]. Thirdly, the sulfonate acid groups in rGO could not only prevent the agglomeration of rGO leading to a high specific surface area for efficient gas adsorption and diffusion, but also selectively absorb NO2 molecules, resulting in the enhancing sensitivity and selectivity of the composites [106,110,111].

3D graphene/MOx hybrids can be also used to detect other gases such as NH_3_, HCHO, H_2_ and so on at room temperature [78,102,103,112,113]. For example, Feng et al. reported 3D rGO encapsulated Co_3_O_4_ nanocrystals fabricated by using the electro-spinning method for room temperature NH_3_ sensing [102]. The 3D rGO/Co_3_O_4_ hybrids showed nanofiber morphology and the Co_3_O_4_ nanocrystals were wrapped by rGO thin layers. The 3D rGO/Co_3_O_4_ nanofiber-based sensors exhibited a p-type semiconductor behavior and showed a high sensitivity (53.6%) and fast response (4 s), recovery rate (5 min) to 50 ppm of NH_3_ at room temperature. In addition, the sensor exhibited good selectivity to some potential interferents such as methanol, ethanol, formaldehyde, acetone, benzene, and methylbenzene. The enhanced room temperature sensing performance was attributed to the unique hierarchical wrapping microstructure and the selective NH_3_ adsorption at both the wrapping layer of rGO and the polarized C-Co^3+^ covalent centers within the nanofibers [102]. In the other case, we recently reported highly effective 3D hybrids sensing films assembled by rGO nanosheets and 3D ZnO flowers to improve the room temperature HCHO sensing properties by using layer-by-layer self-assembled method [103]. The prepared 3D rGO/ZnO films showed higher room temperature sensing performance than both ZnO or rGO-based sensors, which was attributed to the presence of more adsorption sites on the surface of the rGO/ZnO hybrid films and the reduced barrier height for the electron transfer during the gas sensing process [103]. Thus, the construction of 3D architectured graphene/MOx based hybrid structures is a promising strategy to enhance room temperature gas sensing properties.

## 4. Conclusions and Perspectives 

In this review, the recent developments in the construction of 3D graphene/metal oxide-based hybrids, as well as their gas sensing application were discussed. A variety of logical strategies were adapted to design the desired 3D nanohybrids by combing rGO with dimensionally different MOx. In addition, as sensing materials, 3D graphene/metal oxide hybrid-based gas sensors exhibit enhanced room temperature sensing performance owing to their superior high surface area, porosity, functionalized structures and the synergistic effects among them, resulting in the improvement of efficient gas adsorption/diffusion and charge transportation. Therefore, such structure-controlled 3D graphene/metal oxide composites could play very important roles as promising candidates for room temperature hazardous gas sensing. 

However, further efforts are required to achieve green, cost-effective and commercial scale production methods for 3D graphene/metal oxide sensing materials. The controllable fabrication of 3D graphene/metal oxide such as regulation of the number of graphene layers and the achievement for the in-situ growth of MOx on graphene sheets remain unsolved challenges. Furthermore, the unclear interaction mechanisms between building units and gas adsorption/diffusion requires more systematic studies coupled with many fields, such as physics, chemistry, electronics and mathematics.

In the future, the environmentally friendly, facile and industry-scale multistep synthesis of 3D architectured graphene/MOx-based highly-active sensing materials containing controllable porous structures, confined size of MOx and functionalized graphene would be highly desirable. In addition, the development of novel syntheses of 3D graphene/MOx nanostructures is also expected to lead to potential applications in detecting more gases such as CH_4_, C_2_H_2_ and so on at room temperature. We hope that this review will help motivate scientific interest in the controllable fabrication of graphene/MOx-based 3D hybrids nanostructures, as well as in their advanced room temperature gas sensing applications.
